# Towards Wireless Detection of Surface Modification of Silicon Nanowires by an RF Approach

**DOI:** 10.3390/nano12234237

**Published:** 2022-11-28

**Authors:** Florian Requena, Samuel Ahoulou, Nicolas Barbot, Darine Kaddour, Jean-Marie Nedelec, Thierry Baron, Etienne Perret

**Affiliations:** 1 LCIS Laboratory, Grenoble INP, University Grenoble Alpes, F-26000 Valence, France; 2 CNRS, ICCF, Université Clermont Auvergne, Clermont Auvergne INP, F-63000 Clermont-Ferrand, France

**Keywords:** humidity dependency, physicochemical characterization, radar, RF characterization, surface functionalization, silicon nanowires, temperature dependency, wireless measurement

## Abstract

This paper shows the possibility to detect the presence of grafted molecules on the surface of silicon nanowires with a wireless RF radar approach based on the measurement of the backscattered signal of a resonant structure on which the nanowires are deposited. The measured resonance frequency allows the determination of the intrinsic properties related to temperature and humidity variations, which can be related to the presence of the grafted molecules. Several functionalizations of nanowires have been realized and characterized. For the first time, an RF approach is used to detect significant differences related to the presence of grafted molecules on the surface of nanowires. In addition to detecting their presence, the obtained results show the potential of the radar approach to identify the type of functionalization of nanowires. A set of six different grafted molecules (including octadecyltrichlorosilane, ethynylpyrene, N3) was tested and correctly separated with the proposed approach. Various measurements of the same samples showed a good repeatability which made the approach compatible with the possibility of differentiating the molecules with each other by radar reading. Moreover, discussions about the application of such functionalizations are made to increase the sensibility of sensors using a radar approach.

## 1. Introduction

Nowadays, silicon nanowires (SiNWs) are emerging as a promising solution for the development of practical devices due to their fascinating properties (high surface-to-volume ratio, one-dimensional semiconductor materials, modular surfaces) [1,2]. Indeed, SiNWs-based devices are used for many applications such as catalysis, biology, lithium batteries, solar cells, or sensors [3]. For sensor applications, it was reported in [4] that a prior functionalization of silicon nanowires could significantly improve the desired performance. Indeed, the functionalization of silicon nanowires, especially by organic compounds, could enhance their selectivity and their sensitivity and thus leading to high-performance sensors. As an example, Chen et al. demonstrated that carboxylic acid functionalized silicon nanowires improved the electron transfer conductivity [5]. They showed that the sensitivity was three times higher compared to unmodified nanowires. There are several techniques for silicon nanowires functionalization. However, they can be grouped into two main categories. The first approach consists of modifying the native oxide surrounding the nanowires (SiOx/SiNWs). This includes silanization or the reaction of SiNWs with organophosphonates. The second one allows one to modify directly the hydrogen-terminated SiNW (hydrosilylation, arylation, halogenation or alkylation). The modified surface is usually thin. For more details about the functionalization methods, the readers can refer to [4,6].

Regarding the characterization methods of modified silicon nanowires, many approaches are currently used. They are most often specific physicochemical methods available in laboratories working on these topics. In Section 2, a detailed presentation is done of these characterization methods for comparison purpose with the novel approach introduced in this paper. Here, we present an RF characterization approach, without contact, allowing us to detect the presence of grafting of the silicon molecules and even to differentiate the grafted molecules. The main idea is based on the measurement of a high-quality-factor fully passive RF resonant scatterer design on which the nanowires are deposited. The scatterer has an analytical model which takes into account the surrounding temperature and humidity. The principle used, detailed in Section 2, is similar to that of a radar. We measure and analyze the RF field backscattered by the resonator on which the different modified nanowires have been deposited. Based on this measurement, we are interested in determining the device response (resonator with nanowires) through the monitoring of the resonance frequency, as a function of temperature and humidity. It turns out that each molecule grafted on the nanowires interacts with the resonator and induces a different behavior when the temperature and humidity change (note that these differences cannot be highlighted when the temperature and humidity is constant). This behavior can be directly characterized from the measured resonance frequency which will vary differently depending on the grafted molecule. We show that the presence of these molecules which are significantly smaller than the wavelength used for the characterization (there are several orders of magnitude of difference) and therefore do not produce any measurable effect on the backscattered signal in the stationary case (constant temperature and humidity), will, however, have a quantifiable effect when temperature and humidity vary. This effect is highlighted in this paper and is used to differentiate the grafted molecules. These results open the way to the potential of RF using backscattering measurement approaches for chemical substances characterization such as surface-functionalized nanowires. Indeed, it is a nondestructive wireless technique, adapted to smoother surfaces, that can measure areas over several centimeters.

RF techniques have already been applied to characterize the humidity sensing properties of SiNWs [7]. The proposed approach is very far from contact characterization approaches consisting in measuring electrical quantities such as voltages on electronic devices sensitive to the molecules attached to them. Indeed, in [8], it was shown that it was possible to evaluate the surface modification indirectly by using molecular probes either in the gas phase or in the liquid phase. To the best of our knowledge, RF approaches have never been used to characterize functionalized silicon nanowires. In the present work, silicon nanowires were modified using different organic groups. Upon exposure to various temperatures and relative humidity, we defined a Gaussian function which allowed us to distinguish the different groups. In addition, we highlighted the effect of the functionalization on the temperature and humidity sensing capabilities.

## 2. Classical Methods for Surface Analysis

Surface analysis is an unavoidable step after surface modification. Indeed, it makes sense insofar as this allows one to verify the success of the surface modification, understand the provided benefits and ultimately improve its quality. A wide range of physical and chemical surface analysis techniques are available in the literature. They can be classified in two general viewpoints: those that measure the elemental composition or chemical species and those that make area topographic maps. In the first category, most of the tools used for the characterization of silicon nanowires involve spectroscopic methods [9,10], especially infrared (IR) spectroscopy, energy-dispersive X-ray (EDX) and X-ray photoelectron spectroscopy (XPS). IR is a powerful tool for chemical species, molecular structure and defects determination. Generally, attenuated total reflectance (ATR) is used for surface analysis where the sample is mounted on both sides of the trapezoidal IR transparent prism. The sampling depth varies from 10 nm to micrometers. Moreover, EDX is a technique of X-ray spectroscopy that is based on the collection and energy dispersion of characteristic X-rays. This analysis technique is usually associated with electron column instruments such as transmission electron microscope (TEM) or scanning electron microscopy (SEM). The sampling depth change from 0.02 to 1 µm. In addition, an XPS experiment is based on the ejection of electrons, of core levels, from an atom irradiated with a monoenergetic beam of soft X-rays. This technique provides information about the elemental composition, chemical state, depth profiling and mapping. The sampling depth ranges from a few to several nanometers. XPS offers the advantage of being a nondestructive technique. Therefore, it is the most widely used method for surface analysis [11]. For surface profile, scanning tunneling microscope (STM), atomic force microscope (AFM) and SEM are useful tools. STM and AFM offer the possibility to study the film thickness, make topographic imaging or topography, study profilometry and identify the defects in thin films with a sampling depth of less than 0.05 nm. However, STM is based on tunneling current and works only with conducting and semiconducting materials, while AFM can be used to study both insulating and semiconducting materials as well as electrical conducting materials. SEM as well as TEM are microscopy techniques with many advantages: very little sample preparation, no loss of volatile surface deposits and no beam damage problems. The main advantage of SEM is its ability to achieve a very high magnification and resolution of a material surface. Moreover, SEM produces information about the morphology, elemental composition, damages, imaging, or defects of the studied surface with a resolution varying from a few nanometers to a few micrometers. Other techniques, such as electrical resistance and cyclic voltammetry have also found widespread use in investigating the nature of coated surfaces. On the whole, these techniques only give information on small areas.

Compared to optical approaches, RF frequencies are too low (and so is the associated energy) to do imaging of molecules or to interact directly with matter (oscillation, resonance of molecules or atoms...). However, RF approaches are fully compatible to measure the complex permittivity and permeability of materials. In this field many characterization approaches exist, the most developed are those based on the use of transmission lines or cavities in which the material to be characterized is introduced [12,13,14,15]. Wireless RF measurement approaches have also been introduced, for example on similar principles where measurements are done with a VNA and antennas. In such quasi-optical approaches, the transmission and reflection coefficients of a wave that interacts with a plate of the material to be characterized (plate of large size compared to the wavelength of study) allow the permittivity and permeability to be extracted. More recently, a radar approach based on the use of an RF resonator has been used to determine the permittivity of a dielectric [16]. The advantage here is that the method is based on the use of RF resonators, purely metallic, which are positioned on the material to be characterized, which makes its principle of use very flexible and can be used as in here for different applications.

The specificities of these different methods are gathered in Table 1 for comparison. It can be seen that they are very different from the RF approach which is the subject of this article. For example, the principle of operation is very different, insofar as the RF approach is based on the characterization in terms of equivalent permittivity of the behavior of the grafted molecules as a function of temperature and humidity. These possibilities of analysis are very reduced compared to the other methods; however, we show that by this principle, it is possible to have relevant information on the grafted molecules, without a clean room, by using only a radar principle and a climatic chamber.

## 3. Radiofrequency Approach

### 3.1. Principle of the Radar Approach

**Figure 1:**
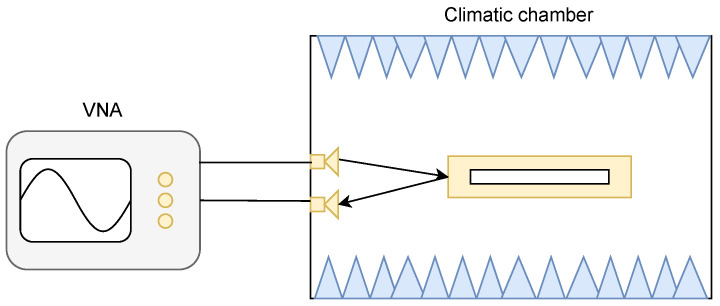
Principle of the measurement of the scatterer’s resonance frequency used to sense temperature and humidity. The climatic chamber allows the control of both temperature and humidity values.

**Figure 2:**
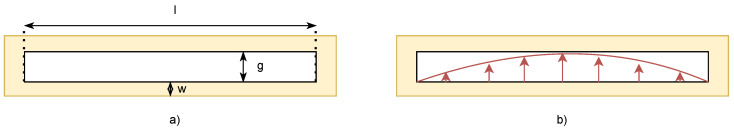
(**a**) Schematic of the loop-sensing resonator. The nanowires are deposited in the center of the gap since it is the spot with the highest E field. (**b**) Illustration in red of the E field concentrated in the loop gap at the resonance.

**Figure 3:**
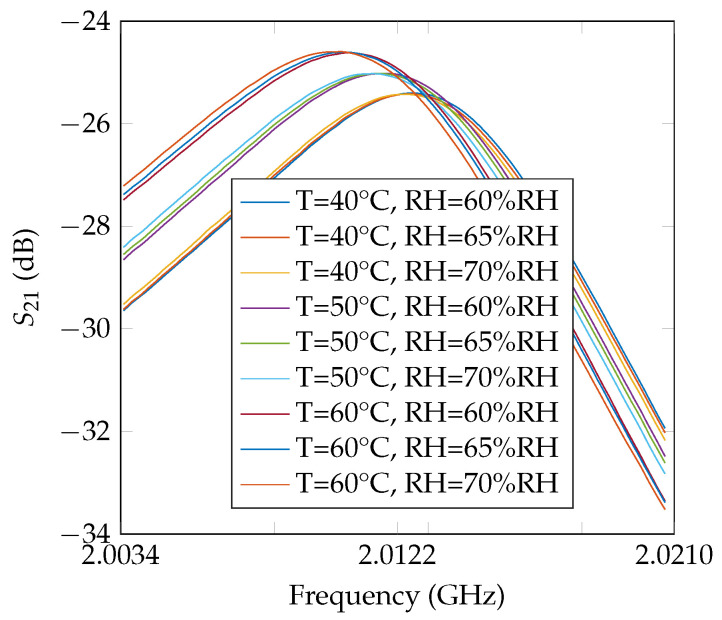
Simulated resonance frequency ($S_{21}$) of a Rogers RO4003C tag for different values of temperature *T* and humidity RH.

The measurement principle based on a radar approach is illustrated in Figure 1. In this paper, loop resonators are used (see Figure 2) but the approach can be used with other resonator shapes [17]. The rectangular loop resonator is prioritized for the fact that at the resonance, the E field is concentrated in a limited region where nanowires can be deposited. The loop resonator also has a high Q-factor and is simple to produce using traditional PCB printing. When a loop resonator is illuminated by an incoming EM field (see Figure 1), the maximum energy backscattered at a temperature *T* and relative humidity RH occurs at its resonant frequency *f* defined in [18]:

(1) $$f\left(T,RH\right)=\frac{c}{2\sqrt{\varepsilon_{eff}\left(T,RH\right)}L\left(T\right)}$$

where *c* is the speed of light in vacuum, $\varepsilon_{eff}\left(T,RH\right)$ is the effective relative permittivity seen by the loop and L(T) the effective length of the loop resonator as defined in [19]. Indeed, the loop resonator can be considered as a transmission line section terminated at both ends by a short circuit (SC). *L* is a function of the physical length of the notch *l* as well as additional lengths considering the presence of short-circuit discontinuities Δl [19]. This resonance frequency *f* as a function of *T* and RH can be developed as [18]:

(2) $$f=\frac{c}{2\sqrt{\varepsilon_{eff}}L_{0}}\left(1-\left(\alpha_{c}+\frac{\alpha_{p}}{2}\right)T-\frac{1}{2}\left[a^{\prime }+\gamma RH\right]\right)$$

where $L_{0}$ is the effective length of the loop resonator at T=0 °C, $\alpha_{c}$ is the thermal expansion coefficient on the effective length, $\alpha_{p}$ and γ are the coefficients for permittivity variations related to temperature and humidity, respectively, and $a^{\prime }$ is a constant that allows the linearization of the variations of nonlinear dielectrics [18]. If the dielectric is linear, then $a^{\prime }=0$. By looking at (2), the resonance frequency is dependent of the temperature *T* and humidity RH and will vary as illustrated in Figure 3. These coefficients are known for common materials and known resonator’s geometries and so (2) can be used to realize temperature and humidity sensors by measuring the resonance frequency of a resonator [18]. When these coefficients are unknown, (2) can be used to extract them with high accuracy. In the case of silicon nanowires, the surface functionalization affects the thermal and humidity sensitivity of the radiofrequency response of the resonator [7] and different functionalizations introduce different unknown values for $\alpha =\alpha_{c}+\alpha_{p}/2$ and γ. Thus, the identification of these coefficients by measurements allows one to distinguish which type of functionalization has been done on nanowires. A multilayer CPS configuration is used where the effective relative permittivity $\varepsilon_{eff}$ of the substrate (in this paper Rogers RO4003C was used) and the superstrate is the nanowires’ deposit [20]. For this reason, the same substrate has to be used with the different nanowires samples to measure the functionalization impact.

### 3.2. Extraction of the Physical Parameters

By considering $f_{ini}$ as the first resonance frequency measured at time $t_{0}$ and the coefficients $a^{\prime }$, α and γ, derived from (2) and using (Equations (16) and (17), [18]), we can have:

(3) $$\frac{f}{f_{ini}}≃1-\frac{a^{\prime }}{2}-\alpha T-\frac{\gamma }{2}RH$$

Notice that for practical reasons, $f_{ini}$ does not need to be at T=0 °C and RH=0%RH [18]. From (3) it is possible to extract the coefficients $a^{\prime }$, α and γ by linear regression applied to the measurement. Indeed, it is possible to measure the resonance frequency corresponding to a set of different values of temperature and humidity for a dedicated loop charged with doped nanowires. The measurement setup presented later in Section 4, as long as we have measured at the same time the temperature and the humidity with an independent electronic sensor, can be used for this extraction.

**Figure 4:**
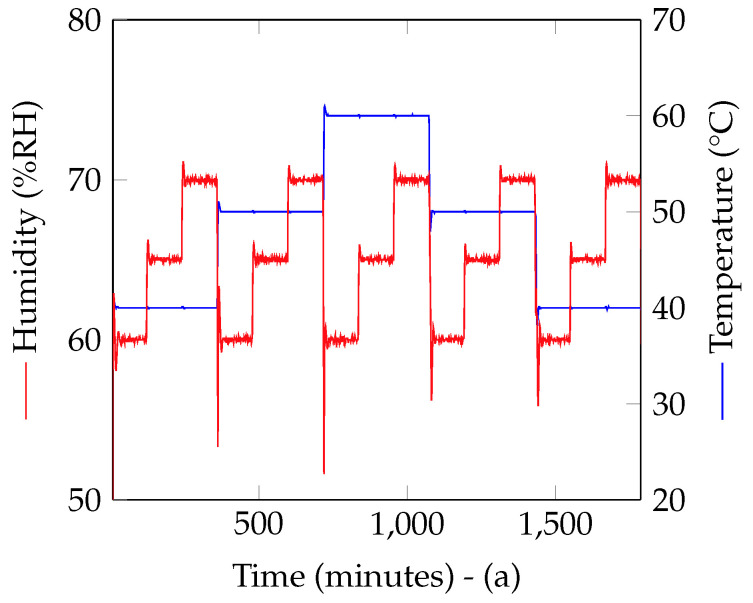
Temperature and humidity measured with an electronic sensor inside the climatic chamber during the measurements.

Measurements in the climatic chamber with the controlled temperature and humidity profiles such as the ones shown in Figure 4 were used to extract the three coefficients. Indeed, once the frequency *f* was measured alongside the temperature *T* and humidity RH, a fit of all the realized measurements was done based on (3) to find the values of α, γ and $a^{\prime }$.

## 4. Fabrication Methods of Nanowires

### 4.1. Experimental Part

#### 4.1.1. Synthesis of Silicon Nanowires

Silicon nanowires growth was performed on a quartz substrate using a horizontal low-pressure chemical vapor deposition (LPCVD) reactor at 600 °C and total pressure of 3 Torr, using ${\mathrm{SiH}}_{4}$ as the precursor gas and hydrogen ($H_{2}$) as the carrier gas. First, the substrate was cleaned in acetone, rinsed with isopropanol and this was immediately followed by the deposition of a 2 nm thick gold layer in a vacuum pressure of $10^{-6}$ Pa. HCl gas was used to inhibit the gold diffusion and two-dimensional (2D) growth. Under such growth conditions, the nanowires exhibited a p-type semiconductor behavior with an estimated density of ionized acceptors of the order of 1015 cm ${}^{-3}$.

#### 4.1.2. Silicon Nanowires Functionalization

In order to form organic silane monolayer covalently attached to a silicon surface, a thin ${\mathrm{SiO}}_{2}$ sheath surrounding the nanowires is required. Indeed, it facilitates silicon nanowires surface modification due to the presence of hydroxyl groups and minimizes the interference from the current flow in the analyte solution. Therefore, all the functionalization operations were carried out on freshly oxidized surfaces. The oxidation process was achieved by soaking the SiNWs (attached to the quartz substrate) in 3:1 $H_{2}{\mathrm{SO}}_{4}$/$H_{2}O_{2}$ solution for 30 min at 80 °C followed by copious rinsing with distilled water. Afterwards, the silicon nanowires were placed in an oven at 70 °C for 30 min.

a. Octadecyltrichlorosilane-modified silicon nanowires (SiNWs-OTS):

The wafer was dipped in 20 µL of OTS in 10 mL of toluene at room temperature for 2 h. After this period, the sample was washed with pure toluene, dried under a gentle stream of nitrogen and placed in an oven at 60 °C for 20 min.

b. Silicon nanowires functionalization with 3-azidopropyltriethoxysilane (SiNWs-$N_{3}$):

The first step of this process was the preparation of 3-azidopropyltriethoxysilane. In 100 mL of acetonitrile, 4 g of 3-chloropropyltriethoxysilane, 2.16 g of sodium azide and 1.3 g of tetrabutylammonium bromide were added. The reaction mixture was placed under a nitrogen atmosphere and stirred at reflux for 24 h. Then, the solvent was removed using a rotavapor under reduced pressure. The residue obtained was diluted in cyclohexane, filtered and washed with ${\mathrm{MgSO}}_{4}$. After removing the solvent afresh, we obtained a crude oil. The oxidized silicon nanowires were immersed in toluene (10 mL) containing 50 µL of 3-azidopropyltriethoxysilane and heated at reflux for 24 h. Afterwards, the sample was rinsed with pure toluene and placed in an oven at 60 °C for 20 min.

c. Ethynylpyrene covalently attached to SiNWs (SiNWs-py):

The pyrene moieties were covalently attached to SiNWs via a click chemistry reaction. First, in a solution of THF (10 mL) of ethynylpyrene (10 mg), we added 3 mL of $H_{2}O$ containing 12 mg of ascorbic acid and 2 mg of ${\mathrm{CuSO}}_{4}$·$H_{2}O$. Then, the azide-functionalized silicon nanowire was immersed in the mixture (in the dark) for 24 h. After that, the nanowires were rinsed carefully with pure THF in order to remove the unreacted ethynylpyrene and placed in an oven at 60 °C for 30 min.

### 4.2. Results and Discussion

We describe hereafter the different analyses that were implemented to ensure the presence of the different functional groups on the nanowires.

#### 4.2.1. Characterization of SiO${}_{2}$-SiNWs

**Figure 5:**
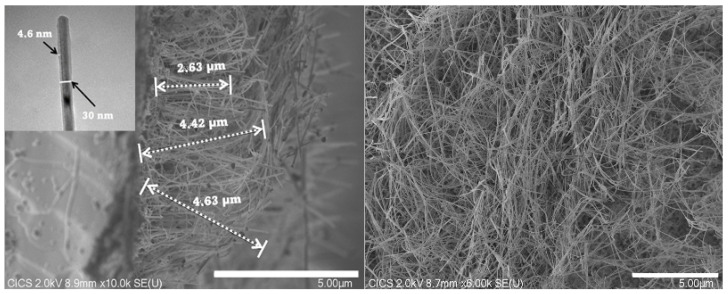
Cross-sectional SEM image of p-type SiO2-SiNWs array. TEM image of SiNW (inset). The insert is a top view picture.

Basically, the CVD synthesis method led to high-purity films and allowed the epitaxial growth of silicon wires [21]. However, the impurities (mainly from gold particles) incorporation into the nanowires were found to induce an anisotropic growth. That is why the pressure of the CVD reactor was lowered in order to reduce unwanted contamination and promoting the epitaxial growth of uniform silicon nanowires. Figure 5 shows the scanning electron microscopy (SEM) of the general morphology of oxidized p-type silicon nanowires. From the several pictures recorded (not shown), the length of SiNWs was found to be between 1 and 5 µm, with an average value of 4 µm. The short SiNWs (≤2 µm) occurred most probably when breaking the substrate. The core diameter of SiO2-SiNWs varied from 20 to 50 nm with an average value of 30 nm. It should be also noted that the silicon nanowires exhibited different growth directions. This behavior was already observed during the silicon growth by CVD, especially for diameters smaller than about 50 nm [22]. Since silicon is known to oxidize easily when exposed at room temperature, we noticed that the ${\mathrm{SiO}}_{2}$ thickness varied around 4 nm (inset).

#### 4.2.2. Octadecyltrichlorosilane-Modified Silicon Nanowires (SiNWs-OTS)

**Figure 6:**
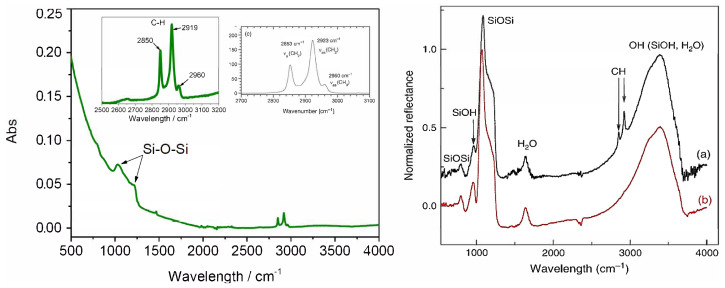
(**a**) Infrared spectrum of an octadecyl-functionalized silicon nanowire; C-H region enlarged, **upper right**; C-H stretching vibration of OTS according to Figure 1C of [23] (inset, **upper left**); (**b**) organosilica films stretching vibration before (**b**) and after (**a**) alkyl groups grafting as shown in Figure 3 from [24].

Oxidized silicon nanowires functionalized with chemically bonded alkyls end groups (OTS) were characterized by means of Fourier transform infrared (FTIR) spectroscopy (Figure 6). The spectrum showed a vibration band of Si-O-Si and Si-O-C between 1000 and 1250 ${\mathrm{cm}}^{-1}$. The presence of the monolayers indicated by two intense peaks obtained at 2850 and 2919 ${\mathrm{cm}}^{-1}$ could be assigned to the symmetric and asymmetric C-H stretching vibrations of CH2, respectively. Finally, the small peak at ≃2960 ${\mathrm{cm}}^{-1}$ could be assigned to the asymmetric C-H stretching vibration of the methyl end group. These results were in good agreement with the literature data as confirmed by Figure 4a’s left inset from [23] and Figure 4b from [24]. It should be briefly recalled that according to theoretical simulations, increasing the length of the alkyl chain increases the van der Waals diameter and hinders the formation of dense alkyl packing [25]. The initial stages in the formation of the alkyl layer involve the linkage between a silane and a hydroxyl group, probably catalyzed by traces of adsorbed surface water (physisorption), which is followed by a condensation reaction, via water consumption, to form a covalent bond (chemisorption). Note that, lateral interactions between long alkyl chains might occur during the physisorption step. A major drawback of long hydrocarbon chains is that they are able to reduce the thermal stability and change the mechanical properties, such as elasticity and hardness. Srinivasan et al. reported that the thermal stability of the OTS monolayer was 150 °C in ambient air and 450 °C in pure $N_{2}$ [26].

#### 4.2.3. Formation of Azide- and Pyrene-Terminated Silicon Nanowires Surfaces

**Figure 7:**
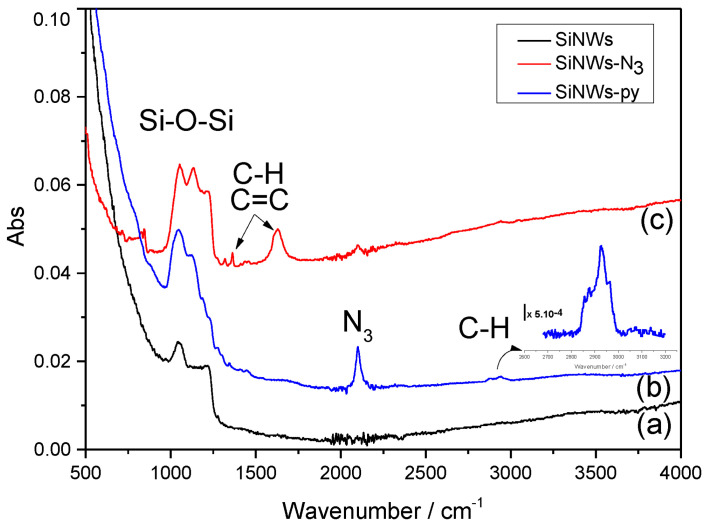
Infrared spectrum of unmodified silicon nanowires (**a**), azide-modified silicon nanowires (**b**), pyrene-modified silicon nanowires (**c**).

As described above, our approach for the preparation of azide-terminated silicon nanowires was based on the silanization of azidopropyltriethoxysilane on oxidized silicon surfaces whereupon the pyrene moieties were covalently grafted via a chemical click reaction. The surface composition and the chemical environment were investigated with FTIR spectroscopy and XPS measurements. Figure 7 shows the different FTIR spectra, from unmodified nanowires to SiNWs-pyrene (SiNWs-py). The unmodified silicon nanowires (a) showed only a vibrational peak of Si-O-Si between 1000 and 1200 ${\mathrm{cm}}^{-1}$ owing to the surface oxidation during the sample treatment. After the silanization of the azide group (b), we could clearly observe the appearance of a new peak at 2100 ${\mathrm{cm}}^{-1}$, characteristic of the $N_{3}$ vibration. The C-H stretching vibrations were located at 2855, 2876 and 2930 ${\mathrm{cm}}^{-1}$ [27]. Briefly recall that the click chemistry reaction is a cycloaddition reaction between alkyne and azide groups [28]. The success of the reaction is typically evidenced by the decrease or disappearance of the $N_{3}$ vibration band after reaction completion [29]. Figure 7 shows a sharp decrease of the azide peak at 2100 ${\mathrm{cm}}^{-1}$ together with the appearance of new peaks at 1612, 1362 and 1319 ${\mathrm{cm}}^{-1}$, typical of the stretching modes of C=C and C-H of pyrene [30]. This latter indicated a successful reaction.

**Figure 8:**
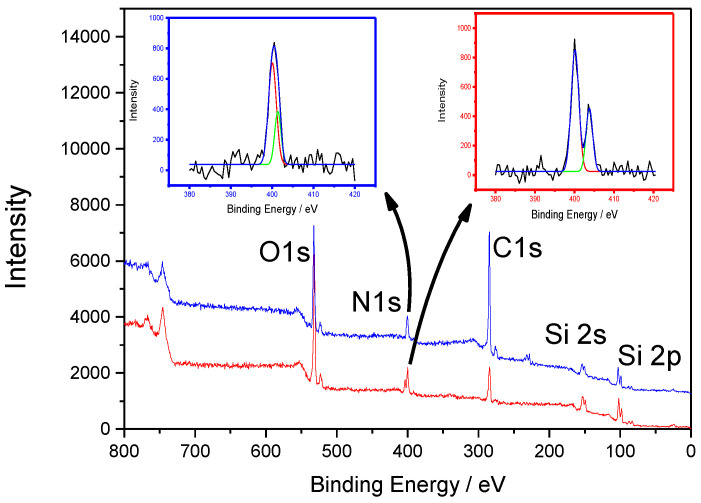
XPS spectra recorded on silicon nanowires modified with azide (red curve) and pyrene groups (blue curve) including enlarged spectra of N1s before and after click chemistry.

XPS analyses were also performed in order to confirm the FTIR observations, especially the incorporation of the $N_{3}$ function and pyrene portion on silicon nanowires surfaces. An XPS survey spectrum of $N_{3}$-SiNWs indicated the presence of C 1s, O 1s, N 1s, Si 2s and Si 2p. We focused on the region of the N1s core level (Figure 8). The peaks at 403.7 eV and 400 eV with a ratio 2:1 were attributable, respectively, to the central electron-deficient nitrogen (-N=N=N) and the two lateral nitrogen atoms (-N=N=N) [31]. However, the same analysis performed after the click reaction with ethynylpyrene showed a single peak which could be fitted into bands at 401.3 and 400.1 eV, attributed to the conversion in azide groups into the 1,2,3-triazole ring [32]. In addition, we observed an increase of the intensity of the carbon peak at 284.5 eV after the click chemistry reaction, which was related to the presence of pyrene moiety on silicon nanowires surfaces.

## 5. Measurements

**Figure 9:**
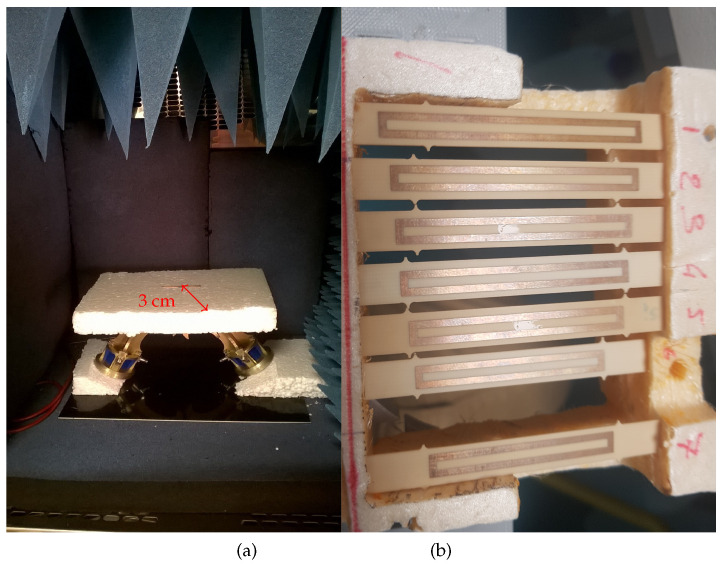
Photo of the measurement bench and tags. (**a**) Setup inside the climatic chamber. (**b**) Copper loop resonators on Rogers RO4003C substrates used for the measurements. Resonances frequencies were 2.013, 2.121, 2.218, 2.287, 2.430 and 2.558 GHz.

The measurement setup and the fabricated tags used in practice are presented in Figure 9.

A climatic chamber VC0018 by Votsch was used to control both temperature and humidity. Absorbers were placed inside the chamber to reduce the reflection level (see Figure 9a). A bistatic antenna configuration was used to increase the isolation between the two ports of the VNA. For the measurements, the following protocol was used: the temperature in the climatic chamber was first set. When the temperature had stabilized, the $S_{21}$ parameter was measured using a VNA. The temperature and humidity inside the chamber were also measured using an external electronic sensor. The measured S-parameters were smoothed to remove any residual measurement noise that may have impacted the determination of the resonance frequency and thus induced an error on the frequency extraction. An IF bandwidth of 10 kHz was also used during the measurements with the VNA with 30,000 points.

This extraction was repeated several times. For each measurement, the entire test bench was disassembled and put back in place. Note also that the deposit of nanowires was redone (i.e., new nanowires deposits were made). Six resonators were measured at the same time (see Figure 9b). One resonator was without nanowires (Rogers) and used to make sure the measurement was done without incident. Indeed, for the specific configuration, the coefficients α and γ were known. They had already been measured and could also be calculated using the provider’s datasheet information [18,19]. These values could be compared with the estimated ones from the new measurement. One resonator was impregnated with silicon nanowires and the four remaining resonators were impregnated with functionalized surface silicon nanowires (respectively, SiNWs-py, pentacene, SiNWs-N3 and SiNWs-OTS). Between each of the measures described here, the nanowires deposits were swapped between the resonators. The process of depositing the nanowires on the resonators is the same as in [7]. With the help of a micropipette, the nanowires mixed in alcohol solutions were manually deposited in the center of the rectangular loop. The alcohol evaporated at ambient temperature, whereas the nanowires remained fixed on the substrate. This zone corresponded to the place where the electric field was maximal at the resonance of the structure. A constant volume of 5 μL was deposited on the resonator using the same concentration solution for each functionalized nanowires. Temperature and humidity profiles were as presented in Figure 4.

### 5.1. Results and Analysis

**Figure 10:**
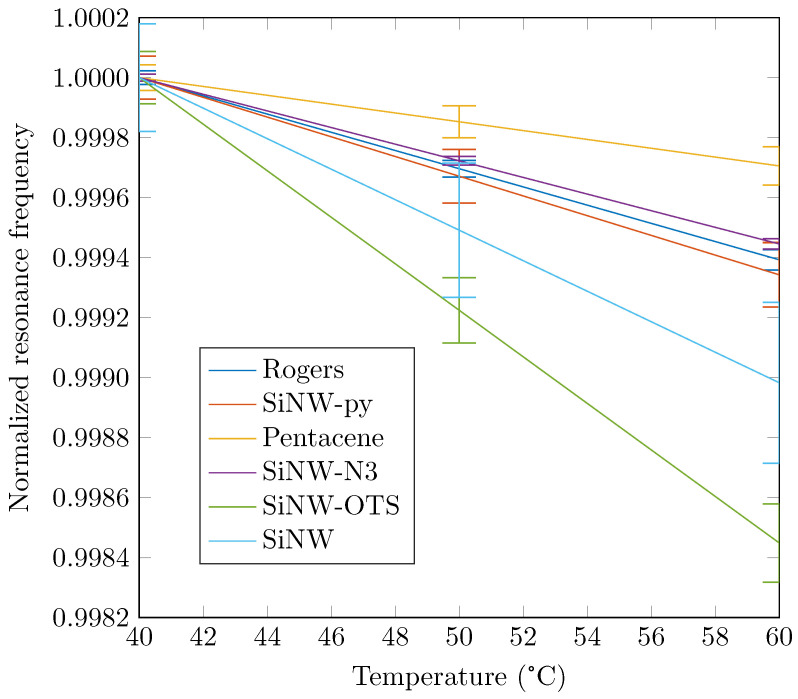
Normalized resonance frequencies for different temperatures at a constant humidity of RH = 60% (normalized by the initial frequency at 40 °C).

**Figure 11:**
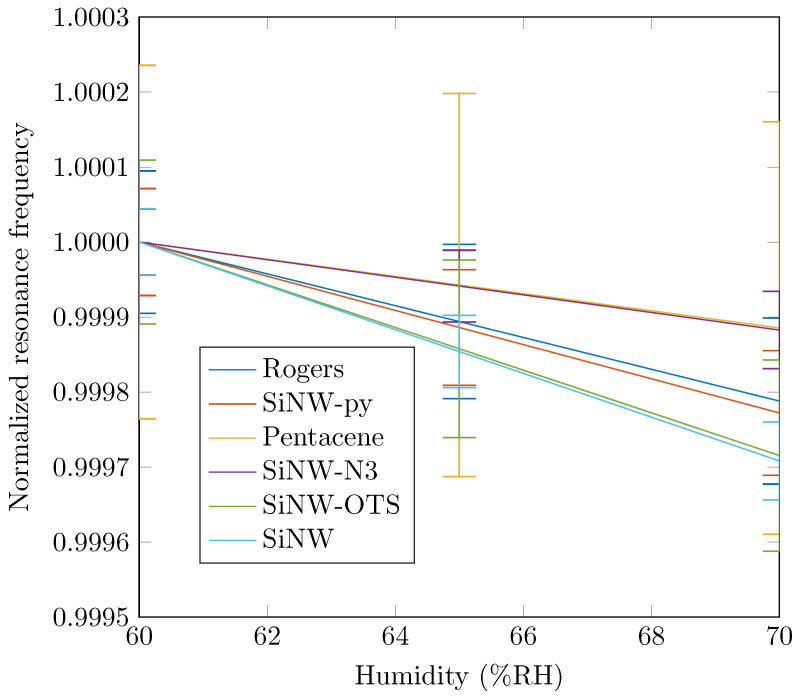
Normalized resonance frequencies for different temperatures at a constant temperature of T = 40 °C (normalized by the initial frequency at 60%RH).

The measurements were repeated ten times; as explained, between each measurement, the resonators were cleaned and the nanowires redeposited. As an example, the first six coefficients α and γ extracted for the sample without nanowires are given in Table 2. These results showed a good repeatability on the extracted coefficients as well as a good estimation compared to the analytical value for α ($\alpha =29.9\times 10^{-6}$—see [19]). Frequency variations as a function of temperature or humidity are plotted in Figure 10 and Figure 11, respectively.

Here, we show the variations as a function of temperature for a single humidity value (60%) and conversely, the variations as a function of humidity for a specific temperature value (40 °C). In Figure 10, we can see that the temperature dependence of the samples, at a fixed humidity, was relatively identical for all the samples except for the undoped nanowires and the SiNWs-OTS. For the latter two, the resonance frequency decreased more rapidly when the temperature increased. Here, we note a maximum variation of the resonant frequency over all the samples of 4 MHz. The evolution of the frequency as a function of humidity is observed in Figure 11. There were more differences in behavior between the six samples, but with smaller variations than for temperature. Here, the maximum frequency difference observed over all samples was 0.5 MHz. The main trend was a decrease in frequency with an increasing temperature. However, pentacene had a very different evolution in that the frequency first increased between 60 and 65% and then decreased for higher humidity values.

In Figure 10 and Figure 11 are also presented error bars corresponding to the standard deviation of each sample observed during the 10 measurements. We can see in Figure 10 that the error intervals due to temperature were small for all samples. The smallest error was obtained for SiNW-N3 while the largest for SiNW. In Figure 11, we can notice that error intervals were wider for humidity especially for the pentacene functionalization. These remarks are discussed later in the manuscript.

**Figure 12:**
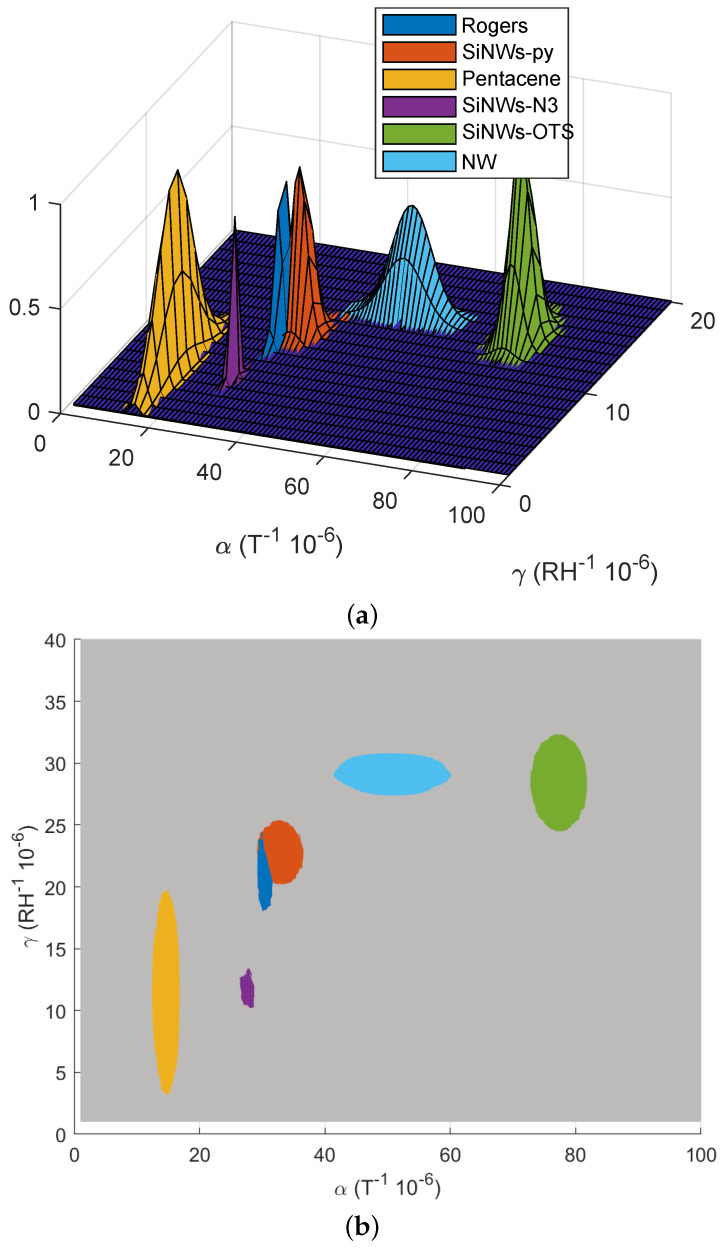
(**a**) Three-dimensional representation of the 6 samples characterized in practice using the climatic chamber and the RF approach. (**b**) Top-view of the Gaussian representation.

In order to be able to collect all the measurement data, but also to ease the reading of the results, a three-dimensional representation was chosen. For each sample, using all the measurements obtained for the different temperature and humidity values, the following Gaussian function *g* was calculated:

(4) $$g\left(\alpha ,\gamma \right)=\exp\left(-\frac{{\left(\alpha -\bar{\alpha }\right)}^{2}}{2\sigma_{\alpha }^{2}}-\frac{{\left(\gamma -\bar{\gamma }\right)}^{2}}{2\sigma_{\gamma }^{2}}\right)$$

where $\bar{x}$ denotes the mean of *x* and $\sigma_{x}$ denotes its standard deviation. The Gaussian functions for each sample are plotted in Figure 12. In addition, the different values of $\bar{x}$ and $\sigma_{x}$ are given in Table 3. The most important observation that can be made directly from Figure 12 is that each functionalization introduced different coefficients α,γ allowing us to differentiate the samples.

If we look in more detail, we can make the following observations. In Table 3 and Figure 12, with regard to the temperature dependence (α), the behavior observed in Figure 10 can be seen again, i.e., pure nanowires and especially SiNWs-OTS were much more temperature-dependent than the other samples. The information that cannot be seen in Figure 10, which represented only a limited number of measurements, is the fact that pentacene had a relatively stable behavior with respect to temperature. Very similar alpha coefficients could be observed between the SiNWs-py and the resonator without nanowires. Finally, when comparing the sample without nanowires and the sample with pure nanowires, a noticeably increased temperature dependence could be observed in the case with nanowires.

Concerning the dependence of the samples on humidity (γ), in accordance with what was already visible in Figure 11, significantly different values were observed in relation to each other but over a smaller range. Indeed, the gamma coefficient varied fairly uniformly between 1.14 and 2.9. The most insensitive sample to humidity was pentacene (followed closely by SiNWs-N3), while the undoped nanowires had a higher gamma of 2.5. Compared to the sample without nanowires, we found as in [7] that the addition of pure nanowires made the resonator sensitive to humidity. This may be of interest for the fabrication of sensors. Generally speaking, we could observe that the variations on the alpha and beta coefficients between the different samples were notable. This showed the very good sensitivity of the measurement, whereas as mentioned in the introduction, the frequency used was of the order of 2.5 GHz, and the volume of nanowires deposited on the samples was extremely small compared to that of the resonator itself.

For a measured ($\alpha_{m},\gamma_{m}$), the likelihood of this sample being silicon nanowires functionalized from the *j*-sample is given by:

(5) $$\mathrm{likelihood}\left(j-\mathrm{sample}\right)=\frac{g_{j}\left(\alpha_{m},\gamma_{m}\right)}{\sum_{i=\mathrm{all}\mathrm{samples}}g_{i}\left(\alpha_{m},\gamma_{m}\right)}$$

**Figure 13:**
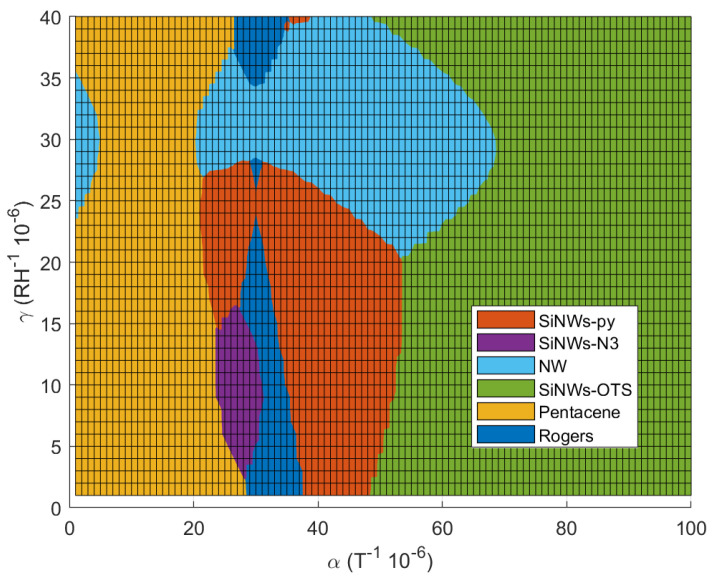
Likelihood representation of the 6 samples.

The likelihood of the six samples is presented in Figure 13. Equation (5) can be used to estimate the percentage of the measured sample belonging to the different samples of the database. The highest probability is given by max(likelihood). This comes from the fact that the likelihood calculation only takes into account the results already present in the database and brings out the sample with the closest criteria to the one measured, even if this new sample does not belong to the database. Note also that (4) can be used to compute the correctness of this sample belonging to the guessed samples from the database. In practice, both formulas (4) and (5) should be used to have a correct estimation on whether or not the sample belongs to the database. For example, a measured $\left(\alpha_{m},\gamma_{m}\right)=\left(80\times 10^{-5},10\times 10^{-5}\right)$ gives 100% likelihood to SiNWs-OTS since it is the only functionalization with $\alpha >60\times 10^{-5}$. However, if we look at Figure 12, we can notice that for this value of $\gamma_{m}$, the sample might not be SiNWs-OTS since $g_{\mathrm{octadecyl}}\left(\alpha_{m},\gamma_{m}\right)$ gives 0% of correctness. The computation of both quantities gives an additional indication, that is to say, the indication on the quality of the measurement: for example if the likelihood is 100% and *g* is 0%, the sample under test can be a new sample which is not from the database or the measurements were dysfunctional.

From Table 3 and Figure 12, we can see that it is possible to distinguish the different functionalizations of silicon nanowires of our samples.

### 5.2. Discussion on the Possibility of Using These Doped Nanowires as Sensors

Other than the detection of the surface functionalization by an RF approach, Figure 12 shows that the sensitivity of the RF response can strongly vary depending on the nanowire functionalization. Numerous sensor applications in the RF domain use the resonance frequency to measure quantities such as temperature or humidity [18,33,34,35]. It has already been shown that nanowires can improve the sensitivity of such sensors by increasing the frequency shifts [7], but the functionalization of the nanowire was never studied. In Table 3, we can notice that the silicon nanowires functionalized by pentacene are a bad candidate for such sensing application because α,γ are smaller than the resonators used without nanowires. Therefore, the frequency shifts will be smaller and harder to measure (see (2) [19]). However, pentacene is a good option to reduce the environment response of temperature and humidity on a resonator and so to improve its response stability towards these variations. On the other hand, SiNWs-OTS are a good candidate for sensor applications. Indeed, both thermal and humidity sensitivities are improved compared to naked Rogers, making the frequency shifts larger. This larger shift does not only allow an easier measurement, but it also improves the accuracy of the sensor [19]. If the coefficient α doubles, the shift in the frequency domain also doubles.

## 6. Conclusions

In this paper we proposed, for the first time, a radar approach for the detection of the surface functionalization of silicon nanowires. The identification of the grafted molecules was done by testing silicon nanowires on a loop resonator and monitoring its resonance frequency during temperature and humidity variations. Measurements were performed using a climatic chamber. The obtained results confirmed the very good potential of the radar approach to identify the functionalization of nanowires, since they were shown to introduced different responses towards humidity and temperature. Moreover, discussions about the application of such functionalizations were made to increase the sensibility of sensors working using the radar approach or to reduce the environmental impact on such resonators.

## Figures and Tables

**Table 1:** Different methods for surface analysis.

Main Information	Technique	Types of Specimen	Main Features	Depth of Analysis
Elemental composition and chemical state	Infrared spectroscopy (IR)	Solid, liquid or gas	High scan speed, high sensitivity, nondestructive technique, unsuitable for sample containing water	10 nm to several micrometer
	Energy-dispersive X-ray (EDX)	Ultrahigh vacuum compatible solids	High detector’s efficiency, ease of use, able to scan areas (∼1 mm^2^) and single spots, destructive analysis	0.02 to 1 µm
	Photoelectron spectroscopy (XPS)	Ultrahigh vacuum (UHV), compatible solids	Can detect almost all elements, most used technique, effective at identifying surface contaminants, samples must be compatible with high-vacuum environment	From 5 to 10 nm
Morphology and imaging	Transmission electron microscope (TEM) or scanning electron microscopy (SEM)	Ultrahigh vacuum compatible solids	Powerful magnification, high-quality images, very expensive and laborious sample preparation	From few nanometers to micrometers
	Scanning tunneling microscope (STM)	All	Three-dimensional profile of surface, operate in large range of temperature (from 0 K to a few hundred degrees Celsius), versatile technique (can be used in air, UHV and water), fragile and expensive, do not work with insulators	<0.03 to 0.05 nm
	Atomic force microscope (AFM)	All	Does not require vacuum and any special treatments; 2D image, 3D surface profile, high resolution, slow scanning rate, single-scan image size (order of micrometers)	<0.03 to 0.05 nm
Permittivity	Radio frequency	Planar surface, flat smooth films, conductors and semiconductors	Nondestructive method, noncontact measurement, wide analysis surface (several centimeters), does not require a clean room.	—

**Table 2:** Extracted coefficients α,γ using (3) for the resonator with no nanowires.

Measure n°	α ($10^{-5}$)	γ ($10^{-5}$)
1	3.026	2.106
2	2.999	2.280
3	3.015	2.030
4	3.148	1.987
5	3.069	1.966
6	3.051	1.904
7	2.938	2.400
8	3.012	2.164
9	3.003	2.266
10	3.074	2.074

**Table 3:** Estimated coefficients α and γ for the different functionalized nanowires.

	$\bar{\alpha }$ ($10^{-5}$)	$\bar{\gamma }$ ($10^{-5}$)	$\sigma_{\alpha }$ ($10^{-7}$)	$\sigma_{\gamma }$ ($10^{-7}$)
No nanowires	3.033	2.118	5.619	15.821
SiNWs-py	3.283	2.276	17.94	11.890
Pentacene functionalized	1.471	1.145	10.65	39.31
SiNWs-N3	2.770	1.173	2.881	7.359
SiNWs-OTS	7.733	2.842	21.761	18.213
Pure nanowires	5.076	2.914	44.762	7.415
